# Choice of outcomes and measurement instruments in randomised trials on eLearning in medical education: a systematic mapping review protocol

**DOI:** 10.1186/s13643-018-0739-0

**Published:** 2018-05-17

**Authors:** Gloria C. Law, Christian Apfelbacher, Pawel P. Posadzki, Sandra Kemp, Lorainne Tudor Car

**Affiliations:** 1 Centre of Population Health Services (CePHeS), Lee Kong Chian School of Medicine, Nanyang Technological University, 11 Mandalay Road, Singapore, 308232 Singapore; 2 Institute of Epidemiology and Preventive Medicine, Regensburg University, Franz-Josef-Strauss-Allee 11, 93053 Regensburg, Germany; 3 Curtin Medical School, Curtin University, 410, Hayman Rd, Bentley, WA 6102 Australia; 4 Family Medicine and Primary Care, Lee Kong Chian School of Medicine, Nanyang Technological University, 11 Mandalay Road, Singapore, 308232 Singapore; 5 Department of Primary Care and Public Health, School of Public Health, Imperial College London, London, UK

**Keywords:** Measurement outcomes, Measurement instruments, eLearning, Randomised controlled trials, Validity, Medical education

## Abstract

**Background:**

There will be a lack of 18 million healthcare workers by 2030. Multiplying the number of well-trained healthcare workers through innovative ways such as eLearning is highly recommended in solving this shortage. However, high heterogeneity of learning outcomes in eLearning systematic reviews reveals a lack of consistency and agreement on core learning outcomes in eLearning for medical education. In addition, there seems to be a lack of validity evidence for measurement instruments used in these trials. This undermines the credibility of these outcome measures and affects the ability to draw accurate and meaningful conclusions. The aim of this research is to address this issue by determining the choice of outcomes, measurement instruments and the prevalence of measurement instruments with validity evidence in randomised trials on eLearning for pre-registration medical education.

**Methods:**

We will conduct a systematic mapping and review to identify the types of outcomes, the kinds of measurement instruments and the prevalence of validity evidence among measurement instruments in eLearning randomised controlled trials (RCTs) in pre-registration medical education. The search period will be from January 1990 until August 2017. We will consider studies on eLearning for health professionals’ education. Two reviewers will extract and manage data independently from the included studies. Data will be analysed and synthesised according to the aim of the review.

**Discussion:**

Appropriate choice of outcomes and measurement tools is essential for ensuring high-quality research in the field of eLearning and eHealth. The results of this study could have positive implications for other eHealth interventions, including (1) improving quality and credibility of eLearning research, (2) enhancing the quality of digital medical education and (3) informing researchers, academics and curriculum developers about the types of outcomes and validity evidence for measurement instruments used in eLearning studies. The protocol aspires to assist in the advancement of the eLearning research field as well as in the development of high-quality healthcare professionals’ digital education.

**Systematic review registration:**

PROSPERO CRD42017068427

**Electronic supplementary material:**

The online version of this article (10.1186/s13643-018-0739-0) contains supplementary material, which is available to authorized users.

## Background

The world is short of 7.2 million healthcare workers, and this will increase to 18 million by 2030 [1]. There are no health services without healthcare professionals, and a direct correlation between the health worker availability in the population, coverage of health services and population health outcomes exists [2]. To further aggravate the problem, the content, organisation and delivery of current medical programmes can fail to equip healthcare workers with knowledge, skills and competencies needed to serve the needs of patients and populations and to meet the changing health needs of the world [3].

Multiplying the number of well-trained healthcare workers through various means and making learning accessible and affordable through innovations and eLearning can potentially solve the shortage of health professionals. The World Health Organisation (WHO) and the United Nations (UN) consider the use of highly innovative, flexible, interactive and adaptive technologies in learning, herein referred to as eLearning, as one of the possible solutions to these problems [4]. eLearning is operationally defined as “the use of electronic media for a variety of learning purposes that range from add-on functions in conventional classrooms to full substitution for the face-to-face meetings by online encounters” [5]. eLearning in medical education involves a broad spectrum of educational interventions characterised by their tools, contents, learning objectives and delivery settings and informed by specific learning theories. These interventions have demonstrated the capability to help healthcare professionals develop knowledge, skills, attitudes and competencies by keeping them actively engaged in learning in a potentially timely, cost-effective and sustainable manner [6]. It has been estimated that over the next couple of years, eLearning will grow 15-fold, accounting for 30% of all educational provision throughout the globe [7].

Randomised controlled trials (RCTs) are considered to be the gold standard research design when evaluating the effectiveness of eLearning educational interventions in improving knowledge, skills, attitudes, competencies and learning satisfaction of healthcare professionals. Systematic reviews provide the best quality evidence synthesis from the RCTs. However, there is a pool of measurement instruments which are used to evaluate the findings of these eLearning trials [8]. Heterogeneity of measurement instruments in systematic reviews leads to the heterogeneity of results, and subsequently, the outcome findings cannot be compared. It has been shown that the eLearning intervention trials lacked certain important outcomes, such as competency-based learning outcomes [9]. There is a lack of agreement as to what are the core outcomes of eLearning in medical education (i.e. primary and secondary outcomes), what measurement instruments are most appropriate for measuring these outcomes and if these measurement instruments have validity evidence to support claims made in relation to learning.

The choice of eLearning outcomes can be influenced by traditions, types of eLearning intervention or the curriculum [10, 11]. Measurement can be defined as the process of determining the extent to which some trait, characteristic, skills or behaviour is associated with a person [12]. The process of measuring eLearning outcomes can be achieved using a wide variety of measurement instruments including multiple choice questions, structured essays or structured direct observations with checklists for ratings and many more [13]. Validity can be defined as “the degree to which evidence and theory support the interpretations of test scores entailed by the proposed uses of tests.” (p. 11) [14]. Validity evidence is necessary to support the interpretation of assessment data.

The intended use of the data collected using the measurement instrument requires sufficient validity evidence. However, two systematic reviews identified scarce reports of validity evidence of measurement instruments used in medical and health profession education. Cook et al. found that only 64% of the 417 studies presented any form of validity evidence in technology-enhanced simulation in health professionals [15]. Ratanawongsa et al. evaluated and reported that only 34.6% of 136 studies in continuing medical education reported any kind of validity or reliability evaluation methods for their measurement instruments [16]. This shortage of measurement instruments with validity evidence undermines the credibility of research results [17] as we cannot ascertain whether the outcomes are adequately measured and interpreted. In the long run, this could affect the quality of healthcare services and potentially patient-related outcomes. Until now, there has been a lack of practical guidelines available for validation of measurement instruments for RCTs in medical education and healthcare research.

The current study is the first step toward ensuring the use of a set of reliable, standardised and validated outcomes and measurement instruments in studies on eLearning for health professionals’ education. The focus and the objective of this review are to determine and map the choice of outcomes, outcome measurement instruments and the prevalence of measurement instruments with validity evidence in eLearning research for pre-registration medical education.

## Methods/design

### Review aims and research questions

This study aims to systematically map and review the outcomes and measurement instruments and to report the prevalence of validity evidence for these measurement instruments in RCTs of eLearning interventions for pre-registration medical education.

We hypothesise that there is a shortage of measurement instruments which provides validity evidence that support learning outcomes such as knowledge, skills, attitudes, competencies, learning satisfaction and patient outcomes in RCTs in eLearning pre-registration medical education. The shortage undermines the credibility of eLearning research leading to the unwarranted interpretation of the results.

In particular, the broad research questions that we aim to answer are:

1. What type of outcomes (e.g. knowledge, skills, attitudes and behaviours) and measurement instruments are used in RCTs of eLearning for pre-registration medical education?
2. What proportion of RCTs on eLearning pre-registration medical education presents validity evidence for the measurement instruments used and how is this evidence reported?

In response to the research questions, we will perform a systematic mapping of research evidence including the types of outcomes, outcome measurement instruments and the proportion of RCTs reporting validity evidence for the measurement instruments used. As part of this systematic mapping review, we will develop a database of eLearning outcome domains and the types of measurement instruments used in relation to each respective domain. We will also search the references of the included RCTs for relevant articles that present validity evidence including psychometric properties of the employed measurement instruments and report their findings. However, in-depth analyses of the quality of validity evidence and synthesis of validity evidence will be performed in another systematic review.

### Eligibility criteria for considering studies for this review

#### Types of studies

We will consider the studies eligible for inclusion if they fulfil the following criteria:

(i) RCTs;
(ii) Involve students participating in pre-registration medical education in any geographical setting or educational setting;
(iii) Evaluate any type of blended or full eLearning method, including a range of eLearning modalities, for example, mLearning, massive open online course (MOOC), online learning, offline learning, virtual reality simulations and digital game-based learning;
(iv) Employ a type of control intervention such as traditional learning, no intervention, other types of eLearning and blended learning.

We adopted the meaning of pre-registration or undergraduate medical education from World Health Organisation by which it means “any type of initial study leading to a qualification that (i) is recognized by the relevant governmental or professional bodies of the country where the study was conducted and (ii) enables its holder primary entry into the healthcare workforce” (p. 11) [4]. Studies will be excluded if they focus on traditional and complementary medicine as defined by WHO [18].

### Search strategies

The search strategy of this review aims to find both published and unpublished studies. No language restrictions will be applied. The search for eligible studies will involve both electronic sources and non-electronic sources.

For the electronic search, the following databases will be searched from January 1990 until August 2017:

- ○ MEDLINE (Ovid)
- ○ EMBASE (Elsevier)
- ○ Cochrane Central Register of Controlled Trials (CENTRAL) (Wiley)
- ○ PSYCINFO (Ovid)
- ○ Educational Resources Information Center (ERIC) (Ovid)
- ○ Cumulative Index to Nursing and Allied Health Literature (CINAHL) (EBSCO)
- ○ Web of Science Core Collection (Thomson Reuters)

We will use the MEDLINE strategy and keywords presented in Additional file 1. This will be adapted to search the other databases. A librarian will be consulted when we adapt the search criteria from MEDLINE to other databases. The reason for selecting 1990 as the starting year for our search is because prior to this year, the use of the computers was limited to very basic tasks. We will search reference lists of all the studies that are deemed to be eligible for inclusion in our review and relevant systematic reviews. We will also search the International Clinical Trials Registry Platform Search Portal and metaRegister of Controlled Trials to identify unpublished trials.

Search results across databases will be merged using the reference management software *EndNote (X7.2.1)* or *Covidence*. Duplicate records of the same report will be removed. Two reviewers will independently examine the titles and abstracts of the records retrieved from the search. The full-text versions of the potentially relevant studies will be retrieved and assessed against the eligibility criteria. Multiple reports of the same study will be linked together, in order to determine if the study is eligible for inclusion. Both the initial screening and the full-text screening will be done independently by two reviewers. Reviewers will correspond with each other to make final decisions on the study eligibility and resolve any disagreements by a discussion with a third reviewer acting as an arbiter if needed. The Preferred Reporting Items for Systematic Reviews and Meta-Analyses (PRISMA) Flow Diagram will be used to report the selection and inclusion of studies [19].

### Data extraction

Two reviewers will independently extract and manage the data for each of the included studies using a structured data recording form. It will include information such as reference of the study, country of the study, the WHO region of the study, name of measurement instrument, description of measurement instrument, types of outcome, assessment category of measurement instruments [13], assessment method of measurement instruments, type of participants, sample size, raters of the instrument, procedure of identifying the raters and training of the raters for the instruments. We will record any sort of validity evidence sources and measurement properties which are reported directly in the articles such as validity, reliability and responsiveness [14, 20]. We will also record any validity evidence which is recorded indirectly, for example, a reference is given to a validation study to a particular instrument. Additional data about outcome measures, instruments and validity evidence will be recorded verbatim if there is a record of validity evidence. If there is more than one outcome measure, relevant details of the second outcome measure will be recorded. The data extraction form will be piloted and amended according to the received feedback. We will contact the study authors for further data in case of missing information. Disagreements between the reviewers will be resolved by discussion. Data will be extracted from all included studies by two reviewers independently. A third reviewer will act as arbiter.

### Data analysis and synthesis

Data will be analysed and synthesised as follows. We will:

(i) Ascertain the types of primary and secondary outcome measurement instruments
(ii) Classify and map the data according to types of outcome (e.g. knowledge, skills, attitudes, satisfaction or competencies); intervention (e.g. online versus offline computer-based eLearning); healthcare profession (e.g. doctors or nurses or allied health professions); types of measurement instruments (e.g. multiple choice questionnaires versus structured direct observation with checklists for rating); and discipline (e.g. anatomy or physiology or pathology)
(iii) Determine the proportion of eLearning RCTs employing measurement instruments with adequate validity in relation to the goal of the measurements (“validity evidence”)

## Discussion

The significance of this systematic mapping review is multifold. First, it will reveal the choice of outcomes, the type of measurement instruments and the proportion of RCTs on eLearning for pre-registration medical education which use measurement instruments with evidence for validity. It will reveal the current state of efforts in this area and advise areas of improvement. Second, our review aims to contribute to the improvement in the quality of reporting, assessment and interpretation of the findings from eLearning research for medical education. eLearning will grow significantly in medical and healthcare education and will in future account for a substantial share of such programmes globally [7]. An understanding of the current state of validation effort among the measurement instruments is a crucial step toward better reporting practices, improved research quality and implementation and effective eLearning interventions supported by reliable results. Efforts have been made to minimise the bias of research methodology and process in clinical trials with established reporting guidelines (e.g. the CONSORT statement). With this review, we intend to support the improvement in credibility and quality of eLearning research field. Lastly, this review will inform other important steps in this research area, for instance, development of reporting guidelines for RCTs on eLearning for health professionals’ education, including a uniform set of recommendations for reporting of measurement instruments.

## Presenting and reporting of the results

We will conduct this review according to the PRISMA standards of quality for reporting systematic reviews.

## Additional files

Additional file 1: Search strings. (DOCX 16 kb)

## Abbreviations

CONSORT: Consolidated Standards of Reporting Trials
PRISMA: Preferred Reporting Items for Systematic Reviews and Meta-Analyses
RCTs: Randomised controlled trials
UN: United Nations
WHO: World Health Organisation
